# Psychometric properties and latent profile analysis of the Nursing Brand Image Scale: a methodological study in the Chinese context

**DOI:** 10.1186/s12912-022-00975-2

**Published:** 2022-09-21

**Authors:** Lu Zhou, Zhao Ni, Judi Allyn Godsey, Khunanan Sukpasjaroen, YuMing Wu, Gao Liu, Thitinan Chankoson, Robert Kallmeyer, EnLi Cai

**Affiliations:** 1 Chakrabongse Bhuvanarth International Institute for Interdisciplinary Studies, Rajamangala University of Technology Tawan-OK, Bangkok, Thailand; 2 School of Nursing, Yunnan University of Chinese Medicine, Kunming, China; 3 School of Nursing, Yale University, New Haven, CT USA; 4 Yale Institution for Global Health, Yale University, New Haven, Connecticut USA; 5 DNP Nursing Faculty, University of Kentucky College of Nursing Lexington, Kentucky, USA; 6 School of Medicine, Yunnan University of Chinese Medicine, Kunming, China; 7 Faculty of Business Administration for Society, Srinakharinwirot University, Nakhon Nayok, Thailand

**Keywords:** Brand Image of Nursing, Nurses, Psychometric Properties, Instrument Translation, China

## Abstract

**Aims:**

To translate the U.S. version of the Nursing Brand Image Scale to Chinese (NBIS-C) and evaluate its psychometric properties when administered to a national sample of Chinese nurses, and identify nursing brand image profiles in Chinese nurses.

**Design:**

A cross-sectional study was conducted to validate the NBIS-C among nurses in China.

**Methods:**

The psychometric properties of the NBIS-C were tested in accordance with the COSMIN checklist. The reliability, validity, and responsiveness of the 42-item NBIS-C were examined in a national sample of 759 nurses recruited from 29 Chinese provinces. Latent Profile Analyses (LPA) were conducted to reveal nurses’ perceptions of the brand image of nursing.

**Results:**

Results of this study demonstrated acceptable validity (content validity, structural validity, and construct validity), reliability (internal consistency and test-retest reliability), adequate responsiveness, and no floor/ceiling effect of the NBIS-C. LPA yielded five subgroups: Integrated, Traditional, Subordinate, Creative and Leader.

**Conclusion:**

The psychometric properties of the NBIS-C are suitable for assessing the image of nursing among Chinese nurses. Future studies with a larger, more diverse sample are recommended. Although the role of nurses in China has evolved, nurses in general have failed to communicate a consistent, positive, and accurate brand image for the nursing profession.

**Supplementary Information:**

The online version contains supplementary material available at 10.1186/s12912-022-00975-2.

## Introduction

The importance of nurses has not been well recognized by the public despite their essential contributions to human health [1]. Traditional stereotypical images of nurses as angels of mercy or subordinates of physicians with minimal education undermine the public image of nurses, reduce the allocation of resources to nursing research, and decrease nurses’ quality of life (Godsey JA, Kallmeyer R, Hayes T: Public Validation of Brand Image of Nursing Scales: Implications for Global Health, unpublished). The stereotypical image of nurses can be commonly seen in the mass media, including magazines, television, and the Internet. For example, prior to the outbreak of the COVID-19 pandemic, nurses in China were often characterized by the media as a group of caregivers who received little education and had no expertise, and therefore, were not highly valued by the Chinese society [2]. The stereotypical images were largely due to the public’s perception that nurses’ work was equivalent to caring and serving others, which did not require expertise or extensive education [3].

After the outbreak of the COVID-19 pandemic, the image of nurses began to be evolved into tireless healthcare providers fighting on the frontline against a pandemic. This heroic view of nurses became a common report in the media and had greatly influenced the previous stereotypical perceptions of nurses [4], thus offering a booster to nurses’ virtuous image [5]. However, the benefits of this media trope on the virtuous image of nurses are projected to be temporary unless an accurate and consistent brand image of the profession is promoted and managed [6]. Highlighting the most virtuous features of nurses but ignoring the intensive professional and intellectual requirements of nurses on their education and training is not only a disservice to the brand image of nursing, but also reducing the attractiveness of the nursing profession to future nurses [7].

Branding is a marketing tool that is used to communicate core values, identify products and services, and positively influence public perception [8]. Intentional efforts to identify and strengthen the nursing brand image are currently underway in the U.S., with the hope to stimulate professional cohesiveness and consistency, enhance the profession's image, and eliminate role ambiguity [9]. The process of effective branding of the nursing profession could result in the conveyance of an image that is relevant, positive, accurate, distinctive, and research based [9]. The brand image could be most effectively communicated through consistent messages and actions over time [10]. A consistent message that highlights the roles and contributions of nurse leaders, scientists, and practitioners is not only desirable for the nursing profession but could also serve as a foundation for institutional strategic plans and college curricula to support the advancement and influence of nurses [9].

A review of literature revealed 11 original scales developed between 1991 and 2021 to measure the image of nursing. Given the paucity of available instruments in the literature, articles that included measures of professional self-concept (or “how nurses feel about themselves as nurses”) were retained [11]. The final list of scales from this review included The Porter Nursing Image Scale (PNIS) [12], the Nursing Image Scale (NIS) [13], the Nursing Attitudes/image Questionnaire (NAQ/NIQ) [14], the BELgian Professional Self-IMAGE Instrument (BELIMAGE) [15], the Professional Self-Concept of Nurses Instrument (PSCNI) [16], the Nurses Self-Concept Instrument (NSCI) [17], the Nurses Self-Concept Questionnaire (NSCQ) [18], the Nurse Self-Description Form (NSDF) [19], the Nursing Brand Image Scale (NBIS), the Nursing's Current Brand Position Scale (NCPBS) and Nursing's Desired Brand Position Scale (NDBPS) [9].

Among all scales examined in this review, the NBIS was the only instrument that incorporated the concept of the brand image of nursing and measured a more comprehensive nursing image. The internal consistency and reliability of the scale were good to excellent in a sample of 286 American Registered Nurses [9].

The importance of the nursing profession has often been overlooked due to inaccurate societal views and outdated stereotypes that negatively influenced nurses’ images. To correct the inaccurate views and stereotypes, nurses and relevant practitioners need to understand the current nursing brand image. However, empirical instruments that measure the comprehensive brand image of nursing are sparse in the literature. And psychometric properties of NBIS Chinese version remain unclear. Moreover, no study has thus far targeted latent profile analysis (LPA) on the brand nursing image. The aims of this study were to translate the U.S. version of the NBIS into Chinese (following the process of the Consensus-Based Standards for the Selection of Health Measurement Instruments [COSMIN] checklist) [20, 21], to evaluate its psychometric properties when administered to a national sample of Chinese nurses, and to identify nursing brand image profiles in Chinese nurses.

## Methods

### Study design and setting

This cross-sectional and methodological study was conducted between July 1, 2021, and November 1, 2021. A non-probability sample of Registered Nurses was recruited from four university-affiliated hospitals located in Southwest China. Snowball sampling was also used to recruit Registered Nurses from other parts of China, covering 29 provinces.

### Participants

Participants were Registered Nurses who had been working in China providing nursing care for greater than 6 months. Interested participants were screened through an online questionnaire and eligible participants provided electronic informed consent prior to accessing the study instruments. A total of 985 nurses completed the screening questionnaire, but those who did not fully complete the questionnaire (*n* = 26) or did not meet the identity verification requirement (*n* = 200) were excluded, resulting in the final analyses of 759 nurses. To ensure reliability and validity, the sample size was estimated based on the recommended 5 to 10 subjects per item of the instrument (the NBIS-C is composed of 42 items) [22]. The sample size of confirmatory factorial analysis (CFA) was estimated based on the G Power package [23]. The close fit and not-close fit were tested in G Power, and the power exceeded 0.99 in both instances. The sample size was between 109 ~ 150 [24]. Thus, a sample of 420 for EFA and 339 for CFA was determined to have adequate power to detect effects.

### Instruments

The Nursing Brand Image Scale (NBIS) was specifically developed to measure nurses’ perceptions of their brand image [9]. The NBIS consists of seven image domains: Strong Interpersonal Skills (4 items), Expert Health-Care Providers and Partners (3 items), Valued By Society (2 items), Qualified Caregivers (6 items), Influential Leaders/Interprofessional Partners (13 items), Qualified for Advanced Nursing Practice (7 items), and Lack Authority/ Professional Identity (7 items). Participants were asked to rate (on a 10-point Likert scale) their level of agreement, and later ranked (top three) each of the 42 items descriptive of the nursing profession. The Chinese version of the NBIS was developed with the permission from the original author, Judi Allyn Godsey. The survey included a socio-demographic questionnaire and one additional measure for comparison of constructs similar to the NBIS: The Nurses Self-Concept Instrument [17]. The Nurses Self-Concept Instrument includes four dimensions and 14 items which have been properly cross-culturally adapted for use in Chinese nurses.

### Translation and cross-cultural adaptation of the NBIS nurse version

According to COSMIN guidelines [20], a Chinese adaptation was carried out, which involves adaptation, not just translation.

#### Translation (from English to Chinese)

Two Chinese bilingual scholars independently translated the original English version of the NBIS; one of the translators (T1) was familiar with the study’s concepts and the nursing environment, the other (T2) was with a medical background.

#### Synthesis

A synthesis of the two translations was conducted, and a consensus was reached to develop a T-12 version.

#### Back translation (from Chinese to English)

Working from the T-12 version of the scale, two English mother-tongue translators who were with psychological backgrounds carried out the back translation and produced B1 and B2 versions.

#### Expert committee review

After the translations, an expert committee reviewed pre-final version with the three translators, the principal investigator (LZ) and the two co-authors (ZN, JG). The role was to consolidate all the translated versions considering four aspects: (1) semantic equivalence, (2) idiomatic equivalence, (3) experiential equivalence, and (4) conceptual equivalence.

#### Pretesting

Twenty undergraduate nursing students were recruited to examine the comprehensiveness, comprehensibility and time to complete the NBIS (approximately 10–15 min). The linguistic and semantic congruence, cultural relevancy, and conceptual equivalences of the Chinese version was confirmed by original NBIS developers and all translators.

### Psychometric analyses

Content validity, floor/ceiling effect, structural validity, construct validity, internal consistency, test–retest reliability, and responsiveness were evaluated based on the COSMIN checklist [25]. Each of these analyses is described in the paragraphs below.

#### Content validity

Item content validity was evaluated via the item content validity index (I-CVI) [26]. Six experts were invited to score every item of the NBIS, including two professors in nursing, two clinical nursing managers, and two professors in management. Six experts were invited to rate the relevance, comprehensiveness, and comprehensibility on each item based on the *COSMIN checklist with a 4-point scale* [27]. Also, ten undergraduate nursing students reviewed the relevance, comprehensiveness, and comprehensibility quantitatively.

#### Floor/ceiling effects

Floor and ceiling effects indicate the extent to which a score is located at the bottom or top of the scale range. The commonly used 25% threshold was adopted to identify the percentage of the sample with the lowest and highest scores overall. Independent sample t-tests were conducted for high and low groups for each item of the NBIS-C [28].

#### Structure validity

Exploratory Factor Analysis (EFA) was analyzed using IBM SPSS Statistics 23.0. Principal component analyses were used to explore the factor structure and unidimensionality of the scale [25]. Following examination of correlation matrices, communalities, and factor loadings, oblique rotation was selected because of the hypothesis of correlations among factors. Confirmatory Factor Analysis (CFA) was performed using the maximum likelihood (ML) method [29]. The Bollen–Stine bootstrap procedure was used to adjust model fit and parameter estimates to accommodate for the lack of multivariate normality [30, 31]. Multi-group CFA was used to test gender differences in the model to ensure the variable was not driving effects (*p* > 0.05, △CFI < 0.01, or △NNFI < 0.05 indicated no significant differences between the two gender groups).

#### Construct validity

Construct validity of the NBIS-C was assessed using factor structure, convergent validity and discriminant validity. The convergent validity was estimated by the Average Variance Extracted (AVE) and Composite Reliability (CR). Values of AVE ≥ 0.50 and CR ≥ 0.70 were considered adequate [32, 33]. Discriminant validity assesses whether the items in a factor are strongly correlated with another factor [32]. The seven‐factor model was computed by correlational analysis and was considered adequate if Correlation Coefficient < Sqrt (AVE).

#### Reliability

Reliability was assessed by internal consistency and stability (test–retest). The internal consistency was assessed using Cronbach's alpha coefficient (α ≥ 0.80) and CR > 0.70 [32]. Total omega was estimated for the overall scale and seven subscales. Total omega values above 0.70 indicate an acceptable level of composite reliability [34]. The NBIS-C was re-tested after 14 days. This criterion was assessed using the Intra-Group Correlation Coefficient (ICC ≥ 0.70) [35].

#### Responsiveness

The Nurses Self-Concept Instrument was used as the standard to compare the validity of the NBIS. Spearman correlation analysis was used for criterion validity and inter-correlations between the items, the factors and the total scale.

### Latent profile analysis

Data analysis was implemented in R 4.1.2 (R Foundation). Latent Profile Analysis (LPA) was performed on all participants with the 7 dimensions of the NBIS-C via tidy LPA-package [36] to identify image classes. The optimal number of classes was determined by Bayesian Information Criterion (BIC) and Akaike Information Criterion (AIC) values. Analyses started with a single class that was compared to six classes. The model fit was assessed until the optimal number of classes was found using the Bootstrap Likelihood Ratio Test (BLRT). Classification performance of the solution was estimated by discriminant analysis and k = tenfold cross-validation based on Gaussian finite mixture modeling [36].

### Ethical considerations

All procedures performed in this study involving human participants were in accordance with the ethical standards of Rajamangala University of Technology Tawan-Ok Ethics Committee and with the 1964 Helsinki declaration and its later amendments or comparable ethical standards. All participants provided their informed consent before taking the survey in the study.

## Results

### Descriptive statistics

Participants in this study included 759 Registered Nurses from 29 provinces residing in China. Demographic information is summarized in Table 1.

**Table 1:** Socio-demographic data (*n* = 759)

Item	n	%
**Gender**		
Female	634	83.52
Male	108	14.17
Non-Binary/Third Gender	17	2.30
**Race**		
Han	622	81.99
Minority	137	18.01
**Age**		
Under 30	407	53.64
31–40	253	33.33
41–50	55	7.28
51–60	38	4.98
61–70	6	.77
Over 70	0	.00
**Nursing Educational Level**		
LPN/LVN	52	6.90
Diploma or Associate Degree in Nursing (ADN)	172	22.61
Baccalaureate Degree in Nursing (BSN)	427	56.32
Master Degree in Nursing—academic (MSN)	41	5.36
Master Degree in Nursing—practice (MSN)	55	7.28
Doctorate of Nursing Practice (DNP)	6	.77
Doctor of Philosophy in Nursing (Ph.D.)	6	.77
**Educational Level**		
Technical Diploma (LPN/LVN)	32	4.21
Diploma or Associate Degree	180	23.75
Baccalaureate Degree	433	57.09
Masters Degree	64	8.43
Practice/Professional Doctorate (DNP, JD, DBA, etc.)	15	1.92
Research Doctorate (Ph.D.)	20	2.68
Other	15	1.92
**Average Household Income**		
0-2499RMB	44	5.75
2500-4999RMB	134	17.62
5000-7499RMB	174	22.99
7500-9999RMB	76	9.96
10,000-12499RMB	87	11.49
12,500-14999RMB	49	6.51
15,000-17499RMB	26	3.45
17,500-19999RMB	15	1.92
Over 20000RMB	41	5.36
Other	113	14.94
**Primary Role In Nursing**		
Nursing Researchers	73	9.58
Clinical First-Line Nurse	480	63.22
Nursing Clinic Nurse	96	12.64
Nursing Educator	55	7.28
Nursing Manager	44	5.75
Nursing Policy Maker	12	1.53
**Geographic Location**		
Yunnan	265	34.87
Sichuan	73	9.58
Guangdong	58	7.66
Chongqing	47	6.13
Hebei	29	3.83
Hubei	23	3.07
Henan	23	3.07
Shandong	23	3.07
Jiangsu	23	3.07
Hunan	20	2.68
Zhejiang	17	2.30
Liaoning	17	2.30
Jiangxi	17	2.30
Jilin	17	2.30
Anhui	15	1.92
Guangxi	12	1.53
Shaanxi	9	1.15
Hainan	9	1.15
Fujian	9	1.15
Shanghai	9	1.15
Tibet	9	1.15
Beijing	6	.77
Shanxi	6	.77
Guizhou	6	.77
Heilongjiang	6	.77
Inner Mongolia	3	.38
Gansu	3	.38
Tianjin	3	.38
Ningxia	3	.38

### Psychometric properties

#### Content validity

The item-level content validity index (I-CVI) was 0.86 ~ 1.00, and the scale-level content validity index (S-CVI) was 0.933. None of the ten nursing students reported confusion or non-comprehension of the items on the NBIS-C.

#### Floor/ceiling effects

Statistically significant differences were found between the high and low groups for all entries (*p* < 0.05, t-value > 3), as detailed in Supplementary Appendix 1.

#### Structural validity

The principal component method of the NBIS-C showed the Kaiser–Meyer–Olkin (KMO) was 0.906 and Bartlett’s Spherical Test was statistically significant (χ^2^ = 6492.449, df = 0.861, *p* = 0.000). Oblique rotation resulted in a seven-factor, 42-item solution that explained 63.66 percent of the variance. Results of exploratory principal component analysis and cross-cultural translation and adaptation are detailed in Table 2.

**Table 2:** Rotated Component Matrix for the NBIS-C (*n* = 420)

		Dimension 1	Dimension 2	Dimension 3	Dimension 4	Dimension 5	Dimension 6	Dimension 7	The Chinese version
		31.94%	9.72%	7.45%	4.68%	3.82%	3.26%	2.76%	
Advanced Nursing Practice									高级护理实践能力
Reliable/Dependable	AN31	.844							可靠/可信
Health Care Providers	AN15	.844							卫生保健提供者^a^
Extensive Training	AN11	.830							广泛的培训
Honest/Integrity	AN19	.815							诚实/正直
Ethical	AN12	.770							道德的
Holistic Approach	AN18	.723							整体观
Advanced Degrees	AN17	.612							高学历
Influential Leaders									领导力
Autonomous	IL2		.805						自主性
Leaders	IL25		.626						领导者
Critical Thinkers	IL7		.594						批判性思考者
Powerful/Decision Makers	IL29		.583						强大/决策者
Intuitive/Thoughtful	IL23		.578						预判/深思熟虑
Influential	IL21		.531						有影响力的
Qualified Caregivers									合格的照护者
Spends Most Time With Patients	QC34			.851					大部分时间与患者在一起
Trusted	QC40			.815					值得信赖
Technological	QC39			.809					技术性的
Skilled	QC33			.774					技能
Patient Centered/Focused	QC27			.643					以患者为中心/专注于患者
Talented	QC36			.534					才华横溢
Lack Authority/Professional Identity									缺乏权威/职业认同
Task Oriented	LA37				.784				以完成任务为主
Physician’s Assistant	LA28				.754				医师助理
White Cap/Uniform	LA42				.747				白帽/制服等刻板印象
Hard to identify from other healthcare workers	LA20				.687				很难从其他医疗工作者中识别
Nurturing/Mothering	LA26				.668				伺候/照料
Subservient	LA35				.583				服从
Female	LA14				.553				女性
Valued By Society/Healthcare									被社会重视^b^
Essential Members of Healthcare Team	VS10					.938			医疗保健团队的重要成员^c^
Health Experts	VS16					.724			健康专家^d^
Diverse Career Options	VS8					.675			多样化的职业选择
Valued by Society/Healthcare	VS41					.657			受到社会/医疗保健行业的重视
Researchers	VS32					.633			研究人员^e^
Teacher/Educator	VS38					.573			教师/教育工作者^f^
Interdisciplinary Partners									跨学科协作
Interprofessional	IP22						.836		跨学科
Professional	IP30						.696		专业的
Collaborators/ Facilitators	IP4						.687		合作者/促进者
Competent	IP6						.651		有能力的
Knowledgeable/ Intelligent	IP24						.615		知识渊博/智慧
Evidence Based Practice	IP13						.499		循证实践
Strong Interpersonal Skills									人际沟通
Empathetic	SIS9							.728	善解人意
Advocates	SIS1							.580	倡导者
Caring/Compassionate	SIS3							.545	关怀/富有同情心
Communicators	SIS5							.433	交流者

Extraction method: principal components analysis

Rotation method: oblique rotation

Rotation converged in 8 iterations

Variance explained: 63.655%

Remark:

a: Reallocated from Expert Health-Care Providers and Partners to Advanced Nursing Practice

b:Merged the dimensions of Expert Health-Care Providers and Partners and Valued By Society

c: Reallocated from Expert Health-Care Providers and Partners to Valued By Society

d: Reallocated from Expert Health-Care Providers and Partners to Valued By Society

e: Reallocated from Advanced Nursing Practice to Valued By Society

f: Reallocated from Advanced Nursing Practice to Valued By Society

**Fig. 1:**
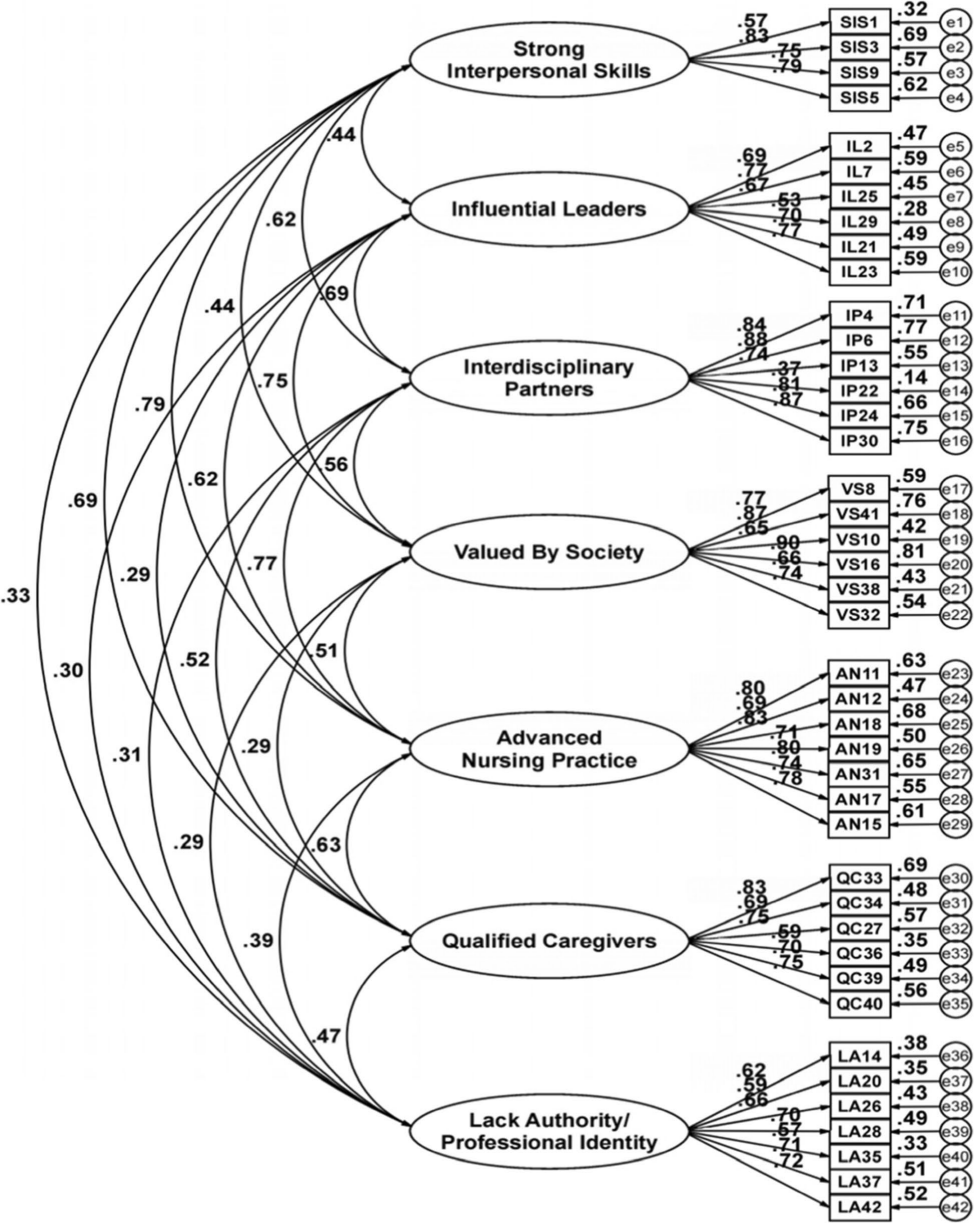
Standardized Path Coefficients (*n* = 339) *|* Note: Seven factor model for measuring the brand image of nursing

Confirmatory Factor Analysis of the seven-factor model was performed on 339 valid questionnaires (Fig. 1) and demonstrated a satisfactory fit to the Chinese nurses’ sample after adjusting model fit with the Bollen–Stine bootstrap p procedure [Bollen-Stine Chi-square = 985.19, x^2^/df = 1.23, GFI = 0.92, CFI = 0.98, TLI = 0.98, IFI = 0.98, RMSEA = 0.03, Standardized RMR = 0.07]. The unstandardized coefficients for the CFA were detailed in Supplementary Appendix 2.

Group differences were determined using multi‐group analysis in CFA [37]. The unconstrained structural model was compared with the constrained structural model, which showed the weights, covariance, and residuals to be equal between the total sample and the male/female sub-samples (*p* > 0.05,△CFI = 0.000, △NNFI = 0.000 ~ -0.014). Results indicate that the measurement model achieved scalar invariance in the male and female groups. Model fit and the resulting comparisons of models are presented in Supplementary Appendix 3 and Supplementary Appendix 4 respectively.

#### Construct validity

The results of convergent validity were adequate except for the Influential Leaders subscale and Lack Authority/Professional Identity subscale (AVE_IL_ = 0.479; AVE_LA_ = 0.429). The results of discriminant validity were adequate except for the Valued by Society and Advanced Nursing Practice dimensions (AVE_VS_ > 0.692; AVE_AN_ > 0.744). Details are shown in Table 3.

**Table 3:** Sqrt (AVE), correlation coefficient matrix, mean, SD, Cronbach’s a, ω, and ICC

	**CR**	**AVE**	**SIS**	**IL**	**IP**	**VS**	**AN**	**QC**	**LA**
SIS	**.829**	**.553**	**.744**						
IL	**.845**	**.479**	.445	**.692**					
IP	**.894**	**.597**	.620	.691	**.773**				
VS	**.896**	**.593**	.436	*.754*	.563	**.770**			
AN	**.907**	**.584**	*.795*	.623	.769	.506	**.764**		
QC	**.866**	**.522**	.693	.290	.518	.290	.629	**.722**	
LA	**.839**	**.429**	.333	.299	.309	.289	.388	.474	**.655**
Mean	**-**	**-**	7.87	6.19	7.35	6.36	7.61	8.03	6.69
SD	**-**	**-**	1.45	1.87	1.76	1.95	1.57	1.50	1.79
Skewness	**-**	**-**	-.87	-.39	-.81	-.32	-1.03	-1.15	-.64
kurtosis	**-**	**-**	.77	-.41	.35	-.56	1.28	1.61	.23
Cronbach’s a-coefficients	**-**	**-**	.80	.84	.87	.89	.90	.86	.83
ω	**-**	**-**	.82	.84	.89	.89	.90	.86	.84
Test–retest (ICC)	**-**	**-**	.68	.76	.77	.71	.74	.72	.63

Notes: *SIS* Strong Interpersonal Skills, *IL* Influential Leaders, *IP* Interdisciplinary Partners, *VS* Valued By Society, *AN* Advanced Nursing Practice, *QC* Qualified Caregivers, *LA* Lack Authority/Professional Identity

ω: McDonald’s hierarchical subscales omega

#### Reliability

The internal consistency of all subscales of the NBIS-C was over 0.70 and the total scale was 0.94. In the 7-factor NBIS-C, all subscale omega values exceeded the threshold 0.70 [38] and the total score was 0.95. However, test–retest evaluation was not adequate for the *Strong Interpersonal Skills* (ICC_SIS_ = 0.68) and *Lack Authority/Professional Identity* (ICC_LA_ = 0.63) subscales. Intra-Group Correlation Coefficient of NBIS-C was 0.73. Data on reliability results are displayed in Table 3.

#### Responsiveness

The Spearman correlation analysis revealed that the correlation coefficient between NBIS-C and the Chinese version NSCI was 0.477 (*p* < 0.01). The correlation coefficients between NSCI and Strong Interpersonal Skills, Influential Leaders, Interdisciplinary Partners, Valued By Society/Healthcare, Advanced Nursing Practice, Qualified Caregivers, and Lack Authority/Professional Identity were 0.403, 0.550, 0,500, 0.417, 0.454, 0.349, and 0.264, respectively (*p* < 0.01). In addition, the scores between factors were positively correlated (*p* < 0.01).

### Latent profile analysis

To better validate the model of nursing brand image and understand how nurses rate their present brand image, models containing six latent classes were estimated and compared. Table 4 presents the fit indices related to the models with an increasing number of latent classes. An improvement was demonstrated in the values of AIC, BIC, and Entropy between models with two to six latent classes. However, the result of the BLRT_p was higher compared to the model with five latent classes in the case of the six-class with a lower entropy solution. This provided some indication that the inclusion of an additional latent class did not provide significant improvement in the model fit. Therefore, a model with five latent classes was retained and selected for further analysis. The five latent classes were labeled as *Subordinate*, *Creative*, *Leader*, *Traditional* and Integrated subgroups.

**Table 4:** Fit indices for the latent class analysis of the NBIS-C factors

Model	Classes	AIC	BIC	Entropy	prob_min	prob_max	n_min	n_max	BLRT_p	Class Probability
1	1	21,421.38	21,486.23	1	1	1	1	1		-
1	2	19,153.5	19,255.41	0.95	0.98	0.99	0.43	0.57	0.01	.42/.58
1	3	18,838.38	18,977.34	0.93	0.92	1	0.16	0.43	0.01	.422/.161/.471
1	4	18,464.98	18,641	0.92	0.84	0.98	0.08	0.42	0.01	.0931/.1546/.3359/.4164
**1**	**5**	**18,361.15**	**18,574.23**	**0.9**	**0.82**	**0.97**	**0.08**	**0.34**	**0.01**	**.0827/.1433/.0926/.3418/.3396**
1	6	18,307.42	18,557.54	0.87	0.75	0.97	0.05	0.34	0.02	.0827/.1433/.0926/.3418/.3396

*AIC* Akaike Information Criteria, *BIC* Bayesian Information Criteria, *LRT* Lo-Mendel-Rubin Adjusted Likelihood Ratio Test

**Fig. 2:**
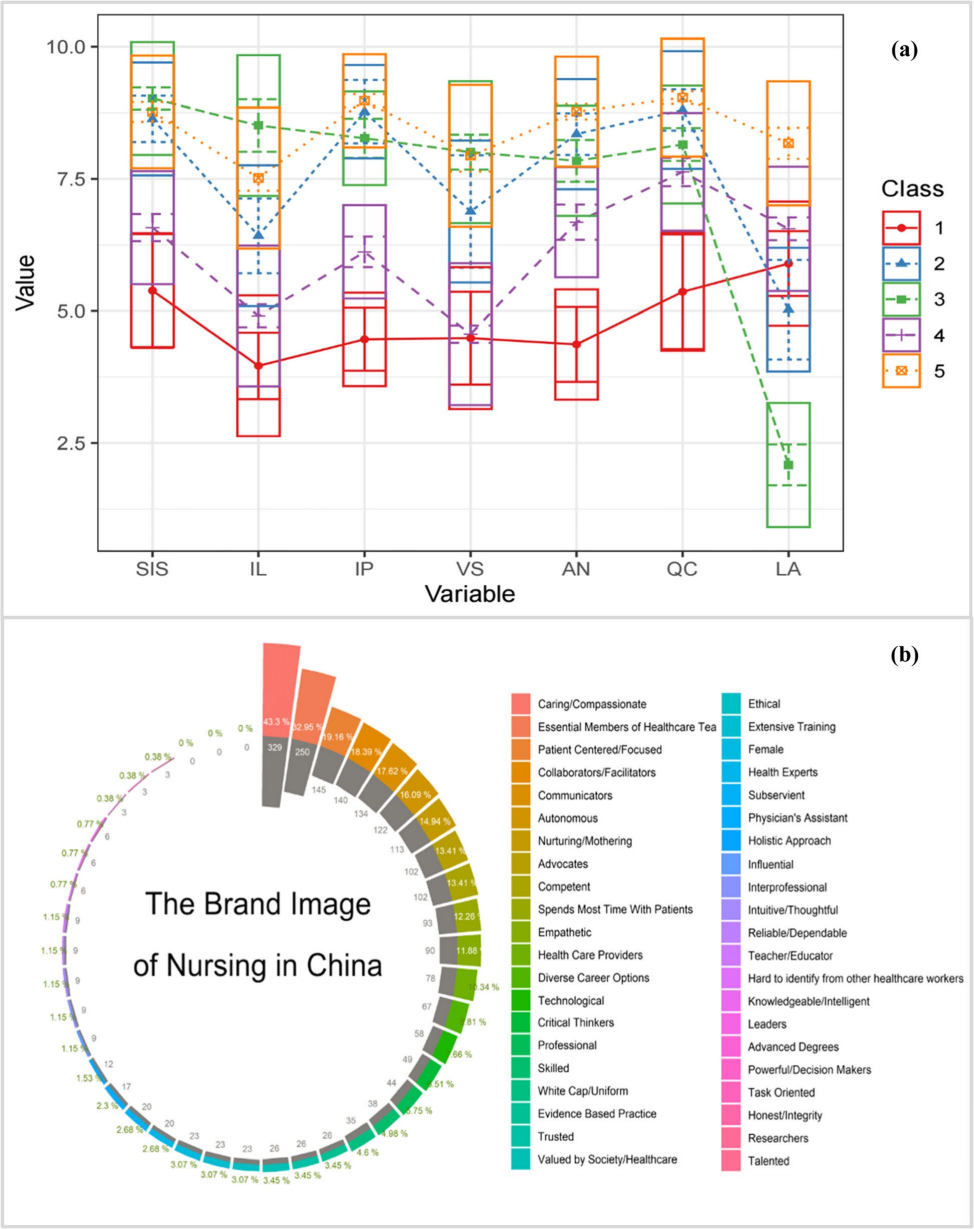
(**a**) Latent class profiles related to the seven NBIS-C dimensions showing the Mean and Standard Deviation | *Notes:* Class 1:*Subordinate*, Class 2: *Creative*, Class 3: *Leader*, Class 4: *Traditional* and Class 5: Integrated. (**b**) Circle Chart of the brand image of Chinese nurses

Around 8% of nurses had low values for all positive brand nursing image domains and high values for negative domains. We identified the subgroup as the least attractive and worst brand image group. 14% percent of nurses' greatest strength is interdisciplinary awareness and advanced nursing practice competencies. In a third detected subgroup, 9% of the nurses, reported the strongest leadership influence and the highest sense of professional identity. The gap between the second and the third subgroups is the largest in the two dimensions of Influential Leaders and Lack Authority/ Professional Identity. Unfortunately, a strong fourth subset of traditional brand image emerged during the analysis. Thirty-four percent of the sample fell into this sub-type, The average score of each dimension also demonstrated that the Chinese nursing brand image is severely underestimated by nurses themselves. Compared to the US data [9]. Finally, 33% percent of Chinese nurses had high scores in all domains. The profile characteristics of the five subgroups based on the average item scores of the seven-factors of NBIS-C are illustrated in (Fig. 2a).

Participants were also invited to choose three words or phrases from NBIS-C that best describe the profession of nursing. The results demonstrated that *Caring/Compassionate, Essential Members of the Healthcare Team*, and *Patient Centered/Focused* reflected the general brand image of nursing in China, shown in (Fig. 2b).

## Discussion

After the NBIS was translated into Chinese, its validity, reliability, and responsiveness were tested based on the COSMIN checklist in a national sample of Chinese Registered Nurses [20]. Results of this study demonstrated acceptable validity (Content validity, structural validity, and construct validity), reliability (internal consistency and test–retest reliability), responsiveness, and no floor/ceiling effect. In the study, we found five categories of the self-perceived brand image among Chinese nurses: Subordinate (category 1), Innovative (category 2), Leader (category 3), Traditional (category 4), and Integrated (category 5).

The reliability of the NBIS-C was found to be acceptable. The results of internal consistency evaluation showed the items of the instrument to be consistent between themselves and predictive of the same construct. Going further than Cronbach’s alpha by testing all McDonald’s omega values, the global results and McDonald’s hierarchical omega subscales confirmed the reliability. In addition, CR values indicated adequate reliability for all subscales. The test–retest stability evaluation showed moderate indices for the Strong Interpersonal Skills and Lack Authority/Professional Identity subscales. A review of the raw data revealed this was due to the variation in the advocate item in the Strong Interpersonal Skills dimension. In Chinese culture, it appears that *advocates* are rarely associated with *interpersonal communication skills in* nursing [39]. The items in the *Lack Authority/Professional Identity* dimension, on the other hand, are more likely to be influenced by self-perception and society [1].

The seven-factor model is different from NBIS in two factors indicated by the results in the EFA. One of the revisions is that the Influential Leaders/Interprofessional Partners subscale in the original NBIS was divided into the Influential Leaders subscale and Interdisciplinary Partners subscale in the NBIS-C. Other than the linguistic usage preference, previous studies found that Influential Leaders and Interprofessional Partners are two different constructs, although some of their features overlap [40]. The two are mutually influencing and independent of each other [41]. The Expert Health-Care Providers and Partners subscale and the items it contains were highly correlated with the dimension of being Valued by Society/Healthcare; therefore, the original NBIS was modified to merge the Expert Health-Care Providers and Partners subscale into Valued by Society/Healthcare subscale.

Overall, principal component factor analysis extracted a seven-factor model consisting of Strong Interpersonal Skills, Influential Leaders, Interdisciplinary Partners, Valued By Society/Healthcare, Advanced Nursing Practice, Qualified Caregivers, and Lack Authority/Professional Identity, which differs slightly in structure from the original study. Therefore, we confirmed the model fit the NBIS-C using confirmatory factor analysis. It is worth noting that the x^2^/df, CFI, TLI, SRMR and RMSEA statistics demonstrated that the seven-factor model offered an acceptable fit with the data collected, indicating that the scale has good structure validity [42, 43]. This changed structure may be more conducive to the cross-cultural adaptation of the scale [43] and enable the evaluation of different brand images in Chinese nurses. Although all items in the factor structure were retained, five items were reallocated in the NBIS-C (Table 2), as indicated by the results in both the EFA and CFA. The difference might result from Chinese cultural and social backgrounds in the development of nursing.

Although all items in the factor structure were retained, five items were reallocated in the NBIS-C (Table 2), as indicated by the results in both the EFA and CFA. The difference might result from Chinese cultural and social backgrounds in the development of nursing. The American Nurses Association (ANA) stated in 1995 that all advanced practice nurses (APN) can make independent or collaborative healthcare decisions [44]. Advanced Nursing Practice has been developed as a professional core curriculum for master's degree students in China [45]. In addition, the outbreak of severe acute respiratory syndrome (SARS) and the COVID-19 pandemic have elevated the value of nurses and demonstrated they are not only the person who gives injections and dispenses medications, but also healthcare providers (Godsey JA, Kallmeyer R, Hayes T: Public Validation of Brand Image of Nursing Scales: Implications for Global Health, unpublished). Thus, item15 (*Health Care Providers*) was considered by most participants to be an important component of advanced nursing practice competencies. The APN needs to assume and be competent in the roles of expert practitioner, educator, researcher, and consultant [46]. The reallocated Item 32 (*Researchers*) and item 38 (*Teacher/Educator*) demonstrated that Chinese nurses' perceptions of advanced nursing practice and their values for society/healthcare are still inconsistent [47]. Participants in this study generally corroborated the seven-factor structure of NBIS-C. Validity in the NBIS-C was found to be nearly identical to the original NBIS. To avoid a biased effect from item 8 (*Diverse Career Options*), future studies could rephrase the wordings in item 32 and item 38 and examine whether these two items can fall back to the original structure as proposed by the NBIS.

The convergent validity presented suitable values for most of the factors, except for the *Influential Leaders* subscale and the *Lack Authority/Professional Identity subscale*, which showed levels below those recommended AVE. Future studies might examine if these two dimensions represent two different brand images of nursing, the traditional and the new. The inconsistency in perceptions of brand image is responsible for the low convergent validity of the two subscales [48, 49]. Regarding the discriminant validity evaluation, there was interpretable identity between *Valued By Society* subscale and *Influential Leaders* subscale, as well as *Strong Interpersonal Skills* and *Advanced Nursing Practice*. A leader ‘s confidence has a positive association with social identity, and their communication skills are essential for advanced nursing practice skills [50, 51]. Generally, the convergent and discriminant validity limitations can be explained due to the high correlations present between the items of the subscales, or due to the item cross‐loadings. Another explanation for these limitations may be related to possible flaws in the scale translation process. However, the cultural adaptation process of the NBIS to Chinese was carefully conducted, and the participants did not report difficulties in understanding any item during the pretest. The factor loadings in *Valued By Society* subscale were also the lowest in the original scale [9].

The main novelty of the research was to generate image profiles in a large Chinese nurse sample using nontheoretical techniques. The different brand images of nursing profiles were performed via LPA to identify subgroups. The LPA revealed five well-interpretable subgroups. These findings demonstrated that the NBIS-C can clearly distinguish between different Chinese nursing brand images. In addition, despite the new and evolving roles in the contemporary nursing practice, the brand image of Chinese nurses is underestimated and inconsistent.

## Strengths, limitations and implications

To our knowledge, this study is the first one to examine the validity, reliability, and responsiveness of the NBIS. Moreover, by uncovering latent subtypes of nursing brand image this study can contribute to the refinement of the NBIS Model. However, some study limitations should be acknowledged. First, the study relied on self-reported data, therefore, it was subject to response biases including social desirability effects. More cross-cultural studies are needed to verify the factor structure of the NBIS-C. Second, for the CFA estimator, robust maximum likelihood (MLR) was conducted for analysis due to the generally less biased standard error estimates and good coverage of the correlations than diagonally weighted least squares (DWLS) [52]. But, DWLS was designed specifically for ordinal data. Thus, DWLS may perform uniformly better than MLR in factor loading estimates. Third, in terms of responsiveness, this study only measured concurrent validity and predictive validity. Finally, the measurement invariance results did not test across demographic characteristics as the sample size to test measurement invariance was small. Future researchers should recruit a larger sample size of nurses from a variety of practice and non-practice settings to evaluate profiles of the brand image of nursing, and explore the differences and relationships across culture and social demography characteristics.

Nurse managers can use the NBIS-C to assess the brand image of nurses in their unique context. Various strategies could be offered to improve nursing’s brand image or to determine if certain features of nursing’s brand image might be predictors of mental health or motivation to improve clinical performance and well-being. To become more influential in the healthcare arena, nurses need to create a more attractive and sustainable brand image that helps retain and energize the current and future workforce [53, 54]. Narrowing the gap between nurses’ current and desired images could be achieved through correcting inaccurate stereotypes, eliminating role ambiguity, and stimulating the professional cohesiveness of the evolving nurse leaders.

## Conclusions

NBIS-C is a valid and reliable scale that can be used to evaluate the brand image of nursing among Chinese nurses. Addressing the gap between nurses’ current and desired images could be achieved through correcting inaccurate stereotypes, eliminating role ambiguity, and stimulating the professional cohesiveness of the evolving nurse leaders.

## Supplementary Information

**Additional file 1:** **Appendix 1.** Independent samplest-test. **Appendix 2.** Path analysis, average variance extracted and composite reliab. **Appendix 3.** Model fit. **Appendix 4.** Model comparisons for Multi-Group CFA

## Data Availability

The original contributions presented in the study are included in the article/supplementary material. Further inquiries can be directed to the corresponding authors.
