# Supplementary material for: Psychometric properties and latent profile analysis of the Nursing Brand Image Scale: a methodological study in the Chinese context
Source: BMC Nurs. 2022 Sep 21;21:259. doi: 10.1186/s12912-022-00975-2 (PMC9490691; doi:10.1186/s12912-022-00975-2)
Supplement: Supplementary file 1 — Additional file 1: Appendix 1. Independent samplest-test. Appendix 2. Path analysis, average variance extracted and composite reliab. Appendix 3. Model fit. Appendix 4. Model comparisons for Multi-Group CFA [file 12912_2022_975_MOESM1_ESM.docx]

**Supplementary Material for Review**

| ***Appendix 1* IndependentSamplest-Test** | | | | | | | | | | |
| --- | --- | --- | --- | --- | --- | --- | --- | --- | --- | --- |
|  |  | Levene’s Test for Equality of Variances | | t-test for Equality of Means | | |  |  |  |  |
|  |  | F | Sig. | t | Degrees of freedom | Sig.(2-sided) | Mean Difference | S.E. | 95% Confidence Interval of the Difference | |
|  |  |  |  |  |  |  |  |  | Lower | Upper |
| Item 1 | Equal Variances Assumed | 14.356 | .000 | 18.501 | 206.000 | .000 | 4.554 | .246 | 4.069 | 5.039 |
|  | Equal Variances Not Assumed |  |  | 18.238 | 178.967 | .000 | 4.554 | .250 | 4.061 | 5.047 |
| Item 2 | Equal Variances Assumed | .153 | .069 | 16.027 | 206.000 | .000 | 4.448 | .278 | 3.901 | 4.995 |
|  | Equal Variances Not Assumed |  |  | 16.022 | 203.856 | .000 | 4.448 | .278 | 3.900 | 4.995 |
| Item 3 | Equal Variances Assumed | 67.780 | .000 | 11.979 | 206.000 | .000 | 2.488 | .208 | 2.079 | 2.898 |
|  | Equal Variances Not Assumed |  |  | 11.557 | 124.302 | .000 | 2.488 | .215 | 2.062 | 2.914 |
| Item 4 | Equal Variances Assumed | 71.614 | .000 | 17.702 | 206.000 | .000 | 3.538 | .200 | 3.144 | 3.932 |
|  | Equal Variances Not Assumed |  |  | 17.083 | 124.745 | .000 | 3.538 | .207 | 3.128 | 3.948 |
| Item 5 | Equal Variances Assumed | 83.573 | .000 | 14.324 | 206.000 | .000 | 3.069 | .214 | 2.647 | 3.492 |
|  | Equal Variances Not Assumed |  |  | 13.823 | 124.774 | .000 | 3.069 | .222 | 2.630 | 3.509 |
| Item 6 | Equal Variances Assumed | 54.130 | .000 | 14.073 | 206.000 | .000 | 3.190 | .227 | 2.743 | 3.637 |
|  | Equal Variances Not Assumed |  |  | 13.606 | 129.073 | .000 | 3.190 | .234 | 2.726 | 3.654 |
| Item 7 | Equal Variances Assumed | 2.533 | .013 | 12.597 | 206.000 | .000 | 3.449 | .274 | 2.909 | 3.989 |
|  | Equal Variances Not Assumed |  |  | 12.574 | 202.262 | .000 | 3.449 | .274 | 2.908 | 3.990 |
| Item 8 | Equal Variances Assumed | 8.278 | .004 | 16.782 | 206.000 | .000 | 4.504 | .268 | 3.975 | 5.033 |
|  | Equal Variances Not Assumed |  |  | 16.578 | 183.845 | .000 | 4.504 | .272 | 3.968 | 5.040 |
| Item 9 | Equal Variances Assumed | 84.063 | .000 | 12.793 | 206.000 | .000 | 2.920 | .228 | 2.470 | 3.370 |
|  | Equal Variances Not Assumed |  |  | 12.326 | 121.079 | .000 | 2.920 | .237 | 2.451 | 3.389 |
| Item 10 | Equal Variances Assumed | 74.329 | .000 | 11.238 | 206.000 | .000 | 2.653 | .236 | 2.188 | 3.119 |
|  | Equal Variances Not Assumed |  |  | 10.873 | 131.038 | .000 | 2.653 | .244 | 2.171 | 3.136 |
| Item 11 | Equal Variances Assumed | 71.807 | .000 | 14.230 | 206.000 | .000 | 3.213 | .226 | 2.768 | 3.658 |
|  | Equal Variances Not Assumed |  |  | 13.718 | 122.409 | .000 | 3.213 | .234 | 2.749 | 3.676 |
| Item 12 | Equal Variances Assumed | 49.505 | .000 | 12.127 | 206.000 | .000 | 2.630 | .217 | 2.202 | 3.057 |
|  | Equal Variances Not Assumed |  |  | 11.731 | 130.459 | .000 | 2.630 | .224 | 2.186 | 3.073 |
| Item 13 | Equal Variances Assumed | 44.085 | .000 | 13.749 | 206.000 | .000 | 3.094 | .225 | 2.651 | 3.538 |
|  | Equal Variances Not Assumed |  |  | 13.307 | 131.834 | .000 | 3.094 | .233 | 2.634 | 3.554 |
| Item 14 | Equal Variances Assumed | 31.739 | .000 | 8.210 | 206.000 | .000 | 2.451 | .299 | 1.862 | 3.040 |
|  | Equal Variances Not Assumed |  |  | 8.072 | 172.228 | .000 | 2.451 | .304 | 1.852 | 3.050 |
| Item 15 | Equal Variances Assumed | 89.634 | .000 | 12.906 | 206.000 | .000 | 2.995 | .232 | 2.537 | 3.453 |
|  | Equal Variances Not Assumed |  |  | 12.436 | 121.283 | .000 | 2.995 | .241 | 2.518 | 3.472 |
| Item 16 | Equal Variances Assumed | 31.846 | .000 | 20.176 | 206.000 | .000 | 4.087 | .203 | 3.688 | 4.486 |
|  | Equal Variances Not Assumed |  |  | 19.686 | 152.435 | .000 | 4.087 | .208 | 3.677 | 4.497 |
| Item 17 | Equal Variances Assumed | 2.598 | .109 | 16.188 | 206.000 | .000 | 3.632 | .224 | 3.190 | 4.074 |
|  | Equal Variances Not Assumed |  |  | 16.018 | 187.741 | .000 | 3.632 | .227 | 3.185 | 4.079 |
| Item 18 | Equal Variances Assumed | 81.348 | .000 | 15.052 | 206.000 | .000 | 3.730 | .248 | 3.241 | 4.218 |
|  | Equal Variances Not Assumed |  |  | 14.504 | 121.401 | .000 | 3.730 | .257 | 3.221 | 4.239 |
| Item 19 | Equal Variances Assumed | 34.401 | .000 | 12.117 | 206.000 | .000 | 2.951 | .244 | 2.471 | 3.431 |
|  | Equal Variances Not Assumed |  |  | 11.897 | 168.939 | .000 | 2.951 | .248 | 2.461 | 3.441 |
| Item 20 | Equal Variances Assumed | 3.525 | .062 | 9.937 | 206.000 | .000 | 3.350 | .337 | 2.685 | 4.015 |
|  | Equal Variances Not Assumed |  |  | 10.067 | 199.898 | .000 | 3.350 | .333 | 2.694 | 4.006 |
| Item 21 | Equal Variances Assumed | 24.721 | .000 | 18.053 | 206.000 | .000 | 4.171 | .231 | 3.715 | 4.626 |
|  | Equal Variances Not Assumed |  |  | 17.656 | 158.577 | .000 | 4.171 | .236 | 3.704 | 4.637 |
| Item 22 | Equal Variances Assumed | 22.194 | .000 | 19.121 | 206.000 | .000 | 4.424 | .231 | 3.968 | 4.880 |
|  | Equal Variances Not Assumed |  |  | 18.744 | 164.769 | .000 | 4.424 | .236 | 3.958 | 4.890 |
| Item 23 | Equal Variances Assumed | 27.004 | .000 | 16.003 | 206.000 | .000 | 3.566 | .223 | 3.126 | 4.005 |
|  | Equal Variances Not Assumed |  |  | 15.582 | 146.891 | .000 | 3.566 | .229 | 3.113 | 4.018 |
| Item 24 | Equal Variances Assumed | 8.493 | .004 | 20.807 | 206.000 | .000 | 4.279 | .206 | 3.874 | 4.685 |
|  | Equal Variances Not Assumed |  |  | 20.454 | 172.067 | .000 | 4.279 | .209 | 3.866 | 4.692 |
| Item 25 | Equal Variances Assumed | 8.015 | .005 | 14.876 | 206.000 | .000 | 4.019 | .270 | 3.486 | 4.551 |
|  | Equal Variances Not Assumed |  |  | 14.753 | 192.230 | .000 | 4.019 | .272 | 3.482 | 4.556 |
| Item 26 | Equal Variances Assumed | 82.124 | .000 | 9.863 | 206.000 | .000 | 2.445 | .248 | 1.956 | 2.934 |
|  | Equal Variances Not Assumed |  |  | 9.510 | 122.786 | .000 | 2.445 | .257 | 1.936 | 2.954 |
| Item 27 | Equal Variances Assumed | 97.207 | .000 | 10.013 | 206.000 | .000 | 2.587 | .258 | 2.077 | 3.096 |
|  | Equal Variances Not Assumed |  |  | 9.666 | 125.451 | .000 | 2.587 | .268 | 2.057 | 3.116 |
| Item 28 | Equal Variances Assumed | 28.022 | .000 | 11.960 | 206.000 | .000 | 3.306 | .276 | 2.761 | 3.851 |
|  | Equal Variances Not Assumed |  |  | 11.705 | 160.380 | .000 | 3.306 | .282 | 2.748 | 3.864 |
| Item 29 | Equal Variances Assumed | 17.230 | .000 | 19.496 | 206.000 | .000 | 4.613 | .237 | 4.146 | 5.079 |
|  | Equal Variances Not Assumed |  |  | 19.137 | 168.167 | .000 | 4.613 | .241 | 4.137 | 5.088 |
| Item 30 | Equal Variances Assumed | 95.295 | .000 | 11.689 | 206.000 | .000 | 2.503 | .214 | 2.081 | 2.925 |
|  | Equal Variances Not Assumed |  |  | 11.273 | 123.176 | .000 | 2.503 | .222 | 2.063 | 2.942 |
| Item 31 | Equal Variances Assumed | 77.552 | .000 | 13.872 | 206.000 | .000 | 2.952 | .213 | 2.532 | 3.371 |
|  | Equal Variances Not Assumed |  |  | 13.382 | 123.795 | .000 | 2.952 | .221 | 2.515 | 3.388 |
| Item 32 | Equal Variances Assumed | 8.542 | .004 | 19.260 | 206.000 | .000 | 4.421 | .230 | 3.968 | 4.873 |
|  | Equal Variances Not Assumed |  |  | 18.918 | 169.895 | .000 | 4.421 | .234 | 3.959 | 4.882 |
| Item 33 | Equal Variances Assumed | 75.269 | .000 | 11.426 | 206.000 | .000 | 2.721 | .238 | 2.252 | 3.191 |
|  | Equal Variances Not Assumed |  |  | 11.037 | 126.911 | .000 | 2.721 | .247 | 2.233 | 3.209 |
| Item 34 | Equal Variances Assumed | 130.712 | .000 | 10.270 | 206.000 | .000 | 2.336 | .227 | 1.887 | 2.784 |
|  | Equal Variances Not Assumed |  |  | 9.858 | 112.842 | .000 | 2.336 | .237 | 1.866 | 2.805 |
| Item 35 | Equal Variances Assumed | 35.211 | .000 | 12.951 | 206.000 | .000 | 3.473 | .268 | 2.944 | 4.002 |
|  | Equal Variances Not Assumed |  |  | 12.620 | 148.984 | .000 | 3.473 | .275 | 2.929 | 4.017 |
| Item 36 | Equal Variances Assumed | 25.890 | .000 | 18.052 | 206.000 | .000 | 4.364 | .242 | 3.888 | 4.841 |
|  | Equal Variances Not Assumed |  |  | 17.666 | 160.292 | .000 | 4.364 | .247 | 3.877 | 4.852 |
| Item 37 | Equal Variances Assumed | 7.486 | .007 | 11.040 | 206.000 | .000 | 3.178 | .288 | 2.611 | 3.746 |
|  | Equal Variances Not Assumed |  |  | 10.988 | 198.581 | .000 | 3.178 | .289 | 2.608 | 3.749 |
| Item 38 | Equal Variances Assumed | 28.419 | .000 | 20.433 | 206.000 | .000 | 4.374 | .214 | 3.952 | 4.796 |
|  | Equal Variances Not Assumed |  |  | 19.975 | 157.543 | .000 | 4.374 | .219 | 3.941 | 4.806 |
| Item 39 | Equal Variances Assumed | 81.603 | .000 | 9.900 | 206.000 | .000 | 2.439 | .246 | 1.953 | 2.924 |
|  | Equal Variances Not Assumed |  |  | 9.562 | 126.748 | .000 | 2.439 | .255 | 1.934 | 2.943 |
| Item 40 | Equal Variances Assumed | 44.022 | .000 | 12.224 | 206.000 | .000 | 2.819 | .231 | 2.364 | 3.273 |
|  | Equal Variances Not Assumed |  |  | 11.862 | 138.226 | .000 | 2.819 | .238 | 2.349 | 3.289 |
| Item 41 | Equal Variances Assumed | 21.466 | .000 | 16.454 | 206.000 | .000 | 4.455 | .271 | 3.922 | 4.989 |
|  | Equal Variances Not Assumed |  |  | 16.200 | 175.933 | .000 | 4.455 | .275 | 3.913 | 4.998 |
| Item 42 | Equal Variances Assumed | 66.694 | .000 | 9.471 | 206.000 | .000 | 2.871 | .303 | 2.273 | 3.468 |
|  | Equal Variances Not Assumed |  |  | 9.188 | 137.635 | .000 | 2.871 | .312 | 2.253 | 3.488 |
|  | | | | | | | | | | |

| ***Appendix 2* Path Analysis, Average Variance Extracted And Composite Reliability** | | | | | | | | | | | |
| --- | --- | --- | --- | --- | --- | --- | --- | --- | --- | --- | --- |
|  |  |  | **Unstd.** | **S.E.** | **t-value** | ***p*** | **Std.** | **SMC/R^2^** | **1-SMC/R^2^** | **CR** | **AVE** |
| Strong Interpersonal Skills | → | SIS1 | 1.000 |  |  |  | .570 | .325 | .675 | .829 | .553 |
|  | → | SIS3 | 1.228 | .103 | 11.890 | *** | .833 | .694 | .306 |  |  |
|  | → | SIS9 | 1.157 | .103 | 11.266 | *** | .754 | .569 | .431 |  |  |
|  | → | SIS5 | 1.248 | .108 | 11.557 | *** | .789 | .623 | .377 |  |  |
| Influential Leaders | → | IL2 | 1.000 |  |  |  | .686 | .471 | .529 | .845 | .479 |
|  | → | IL7 | 1.059 | .076 | 13.960 | *** | .767 | .588 | .412 |  |  |
|  | → | IL25 | .967 | .078 | 12.402 | *** | .671 | .450 | .550 |  |  |
|  | → | IL29 | .877 | .088 | 9.969 | *** | .531 | .282 | .718 |  |  |
|  | → | IL21 | .951 | .074 | 12.929 | *** | .703 | .494 | .506 |  |  |
|  | → | IL23 | 1.017 | .073 | 13.956 | *** | .766 | .587 | .413 |  |  |
| Interdisciplinary Partners | → | IP4 | 1.000 |  |  |  | .842 | .709 | .291 | .894 | .597 |
|  | → | IP6 | 1.060 | .046 | 22.909 | *** | .877 | .769 | .231 |  |  |
|  | → | IP13 | .829 | .047 | 17.706 | *** | .743 | .552 | .448 |  |  |
|  | → | IP22 | .482 | .063 | 7.642 | *** | .371 | .138 | .862 |  |  |
|  | → | IP24 | .976 | .048 | 20.259 | *** | .813 | .661 | .339 |  |  |
|  | → | IP30 | .995 | .044 | 22.469 | *** | .866 | .750 | .250 |  |  |
| Valued By Society | → | VS8 | 1.000 |  |  |  | .770 | .593 | .407 | .896 | .593 |
|  | → | VS41 | 1.105 | .057 | 19.349 | *** | .874 | .764 | .236 |  |  |
|  | → | VS10 | .742 | .054 | 13.641 | *** | .650 | .423 | .578 |  |  |
|  | → | VS16 | 1.114 | .056 | 19.955 | *** | .897 | .805 | .195 |  |  |
|  | → | VS38 | .743 | .054 | 13.825 | *** | .657 | .432 | .568 |  |  |
|  | → | VS32 | .910 | .058 | 15.779 | *** | .737 | .543 | .457 |  |  |
| Advanced Nursing Practice | → | AN11 | 1.000 |  |  |  | .796 | .634 | .366 | .907 | .584 |
|  | → | AN12 | .755 | .050 | 14.983 | *** | .686 | .471 | .529 |  |  |
|  | → | AN18 | 1.088 | .057 | 19.006 | *** | .826 | .682 | .318 |  |  |
|  | → | AN19 | .798 | .051 | 15.546 | *** | .706 | .498 | .502 |  |  |
|  | → | AN31 | .987 | .054 | 18.325 | *** | .803 | .645 | .355 |  |  |
|  | → | AN17 | 1.006 | .061 | 16.570 | *** | .743 | .552 | .448 |  |  |
|  | → | AN15 | .930 | .053 | 17.606 | *** | .779 | .607 | .393 |  |  |
| Qualified Caregivers | → | QC33 | 1.000 |  |  |  | .830 | .689 | .311 | .866 | .522 |
|  | → | QC34 | .755 | .050 | 15.103 | *** | .690 | .476 | .524 |  |  |
|  | → | QC27 | .934 | .055 | 16.890 | *** | .752 | .566 | .434 |  |  |
|  | → | QC36 | .865 | .069 | 12.479 | *** | .590 | .348 | .652 |  |  |
|  | → | QC39 | .803 | .052 | 15.321 | *** | .698 | .487 | .513 |  |  |
|  | → | QC40 | .842 | .050 | 16.828 | *** | .750 | .563 | .438 |  |  |
| Lack Authority/  Professional Identity | → | LA14 | 1.000 |  |  |  | .619 | .383 | .617 | .839 | .429 |
|  | → | LA20 | 1.098 | .111 | 9.920 | *** | .588 | .346 | .654 |  |  |
|  | → | LA26 | 1.031 | .096 | 10.785 | *** | .656 | .430 | .570 |  |  |
|  | → | LA28 | 1.247 | .111 | 11.268 | *** | .697 | .486 | .514 |  |  |
|  | → | LA35 | .984 | .101 | 9.734 | *** | .574 | .329 | .671 |  |  |
|  | → | LA37 | 1.248 | .109 | 11.444 | *** | .712 | .507 | .493 |  |  |
|  | → | LA42 | 1.312 | .114 | 11.535 | *** | .721 | .520 | .480 |  |  |
| CR:Composite Reliability  AVE:Average Variance Extracted | | | | | | | | | | | |

| ***Appendix 3* Model fit** | | | | | | | | |
| --- | --- | --- | --- | --- | --- | --- | --- | --- |
| **Models** | x2 | Chi2/DF | **GFI** | **IFI** | **TLI** | **CFI** | **RMSEA** | **SRMR** |
| Model 0 | 3733.466 | 2.339 | .642 | .715 | .687 | .710 | .073 | .074 |
| Model 1 | 3772.180 | 2.317 | .640 | .712 | .692 | .709 | .073 | .074 |
| Model 2 | 3804.457 | 2.305 | .637 | .709 | .695 | .706 | .073 | .075 |
| Model 3 | 3922.563 | 2.333 | .625 | .693 | .689 | .693 | .073 | .076 |
| Model 0=Unconstrained structural model | | | | | | | | |
| Model 1=Measurement weights’ invariant | | | | | | | | |
| Model 2=Measurement weights' and Structural covariances' invariant | | | | | | | | |
| Model 3=Measurement weights', Structural covariances' and Structural residuals' invariant | | | | | | | | |

| ***Appendix 4* Model comparisons for Multi-Group CFA** | | | | | |
| --- | --- | --- | --- | --- | --- |
| **Comparison** | **△x^2^** | **△df** | **△CFI** | **△NNFI** | ***P*** |
| Model 0 VS. Model 1 | 38.714 | 35 | .000 | -.008 | .306 |
| Model 0 VS. Model 2 | 70.991 | 63 | .000 | -.014 | .229 |
| Model 0 VS. Model 3 | 189.097 | 105 | .000 | -.014 | .000 |
|  |  |  |  |  |  |
| Model 1VS. Model 2 | 32.276 | 28 | .000 | -.006 | .263 |
| Model 1VS. Model 3 | 150.382 | 70 | .000 | -.006 | .000 |
|  |  |  |  |  |  |
| Model 2VS. Model 3 | 118.106 | 42 | .000 | .000 | .000 |
| Model 0=Unconstrained structural model | | | | | |
| Model 1=Measurement weights’ invariant | | | | | |
| Model 2=Measurement weights' and Structural covariances' invariant | | | | | |
| Model 3=Measurement weights', Structural covariances' and Structural residuals' invariant | | | | | |
